# Peripheral Neuropathy During Concomitant Administration of Proteasome Inhibitors and Factor Xa Inhibitors: Identifying the Likelihood of Drug-Drug Interactions

**DOI:** 10.3389/fphar.2022.757415

**Published:** 2022-03-14

**Authors:** Long Meng, Jing Huang, Feng Qiu, Xuefeng Shan, Lin Chen, Shusen Sun, Yuwei Wang, Junqing Yang

**Affiliations:** ^1^ Key Laboratory of Biochemistry and Molecular Pharmacology, Department of Pharmacology, Chongqing Medical University, Chongqing, China; ^2^ Department of Pharmacy, The First Affiliated Hospital of Chongqing Medical University, Chongqing, China; ^3^ Department of Respiratory and Critical Care Medicine, The First Affiliated Hospital of Chongqing Medical University, Chongqing, China; ^4^ Department of Pharmacy, Chongqing Health Center for Women and Children, Chongqing, China; ^5^ Department of Pharmacy Practice, College of Pharmacy and Health Sciences, Western New England University, Springfield, MA, United States; ^6^ Department of Pharmacy, Xiangya Hospital Central South University, Changsha, China; ^7^ Chongqing University Cancer Hospital, Chongqing, China

**Keywords:** proteasome inhibitors, peripheral neuropathy, factor Xa inhibitors, drug-drug interactions, pharmacovigilance

## Abstract

**Backgrounds:** Proteasome inhibitors (PI) cause toxic peripheral neuropathy (PN), which is one of the dose-limiting adverse events of these treatments. Recent preclinical studies find that factor Xa inhibitor (FXaI), rivaroxaban, promotes PN in animals receiving oxaliplatin. Cancer patients can receive combined therapy of PI and FXaI. This study aimed to identify and characterize the interaction signals for the concomitant use of PI and FXaI resulting in PN.

**Methods:** Reports from the United States FDA Adverse Event Reporting System (FAERS) were extracted from the first quarter of 2004 to the first quarter of 2020 for analysis. The Standardized Medical Dictionary for Regulatory Activities (MedDRA) query was used to identify PN cases. We conducted an initial disproportionality investigation to detect PN adverse event signals associated with the combined use of PI and FXaI by estimating a reporting odds ratio (ROR) with a 95% confidence interval (CI). The adjusted RORs were then analyzed by logistic regression analysis (adjusting for age, gender, and reporting year), and additive/multiplicative models were performed to further confirm the findings. Additionally, subset data analysis was performed on the basis of a single drug of PI and FXaI.

**Results:** A total of 159,317 adverse event reports (including 2,822 PN reports) were included. The combined use of PI and FXaI was associated with a higher reporting of PN (RORadj = 7.890, 95%CI, 5.321–11.698). The result remained significant based on additive/multiplicative methods. The observed association was consistent in the analysis restricted to all specific PI agents (bortezomib and ixazomib) and FXaI (rivaroxaban), except apixaban.

**Conclusion:** Analysis of FAERS data identified reporting associations of PN in the combined use of PI and FXaI, suggesting the need for more robust preclinical and clinical studies to elucidate the relationship.

## Introduction

Proteasome inhibitors (PI) have transformed the treatment of hematologic malignancies, especially multiple myeloma, and have become the mainstay of therapy in the last 10 years. Unfortunately, PIs play a role in producing cardiotoxicity and neurotoxicity. Peripheral neuropathy (PN) is one of the major dose-limiting adverse events (AE) of PIs (Schlafer et al., 2017; Gavazzoni et al., 2018). PN causes dysfunction in the sensory, motor, or autonomic nervous systems leading to numbness, allodynia, tingling, paresthesia, and dysesthesias (Cavaletti and Zanna, 2002). The overall incidence of PN induced by bortezomib, the first approved PI, ranged from 31 to 64% in clinical trials (Velasco et al., 2010). Neurotoxicity of grade ≥3 ranged from 7 to 15% (Ruschak et al., 2011). PN can cause delay, reduction in doses, or even interruption of treatment. These treatment effects can affect tumor progression, increase morbidity or economic burden, and reduce patient quality of life (Argyriou et al., 2012; Mols et al., 2013).

Venous thromboembolism (VTE) is a common complication in patients with multiple myeloma, as more than 10% of these patients will develop VTE during the course of their disease (Kristinsson, 2010). Therefore, antithrombotic agents are needed for the prophylaxis and treatment of VTE. In addition to low-molecular-weight heparin (LMWH) and warfarin, factor Xa inhibitors (FXaI) have become favorable choices for anticoagulation in recent years. These inhibitors can be administered orally and do not require blood monitoring at standard doses. As a result, more patients with multiple myeloma are receiving FXaI. Therefore, more patients receive concomitant treatment of PI and FXaI in clinical practice (Swan et al., 2018). However, a recent experimental study found that anticoagulants, such as rivaroxaban and warfarin promote oxaliplatin-induced PN in animals. Furthermore, rivaroxaban can reverse the anti-PN effect of thrombomodulin in animals treated with oxaliplatin (Tsubota et al., 2019). Given these findings, we hypothesized a possible association with PN development in patients receiving combination therapy of PI and FXaI.

Clinical trials or meta-analyses of randomized controlled trials are valuable ways to assess the safety of drugs (Massari et al., 2020; Rizzo et al., 2020). On the other hand, the United States Food and Drug Administration (FDA) Adverse Event Reporting System (FAERS) database collects voluntary AE reports of post-marketed drugs submitted by manufacturers, healthcare professionals, and consumers from United States and non-US countries. Compared to other international databases for spontaneous AE reporting, FAERS has several distinctive characteristics, including the heterogeneous catchment area (to broaden the generalization of findings), the public access to raw data that can be downloaded in a format suitable for customized analysis (Antonazzo et al., 2020). Data mining from the FAERS pharmacovigilance source can be utilized to evaluate drug safety, such as identifying rare or new AEs (Meng et al., 2021) and quantitatively detecting drug-drug interactions (DDIs) (Oshima et al., 2018; Antonazzo et al., 2020). The present study aimed to detect safety signals between the concomitant use of PI and FXaI (both as a drug class and as a single agent) and the appearance of PN.

## Materials and Methods

### Data Acquisition and Preprocessing

Relevant data from the first quarter of 2004 to the first quarter of 2020 were downloaded from the FAERS database. OpenVigil FDA was used to perform disproportionality analyses for data acquisition and preprocessing. OpenVigil FDA is a novel web-based pharmacovigilance analysis tool that uses the openFDA online interface to access the drug-event dataset from FAERS (Böhm et al., 2012; Böhm et al., 2016). It is widely utilized in pharmacovigilance research (Huang et al., 2020a; Huang et al., 2020b; Böhm et al., 2021). OpenVigil only operates on cleaned data by eliminating duplicates or reports with missing data (Oshima et al., 2018).

### Identification of Adverse Events

AE reports in FAERS are coded through the MedDRA of Preferred Terms (PTs). MedDRA version 24 was used to classify AEs in this study. Standardized MedDRA Queries (SMQs) are groupings of MedDRA terms, ordinarily at the PT level, that relate to a defined medical condition or area of interest. SMQs usually include two PT categories, “narrow” scope and “broad” scope. The narrow search consists of terms without any reasonable doubt related to a selected event. The broad search contains terms of the narrow one and terms related to an event of interest, but there is a degree of uncertainty. Consequently, the narrow SMQ “peripheral neuropathy” (such as neuritis) is more specific, while the broad SMQ “peripheral neuropathy” (such as hypoaesthesia) is more sensitive (Cirmi et al., 2020). Therefore, for this study, the narrow search for SMQ “peripheral neuropathy” was used (Supplementary Table S1).

Because DDIs were evaluated, no distinction was made between suspected and non-suspected drug roles. Therefore, all medications were included for analysis (Yue et al., 2014). As a result, cases were identified to assess the occurrence of PN in patients who received a combination therapy of PI and FXaI. Data were extracted using MedDRA SMQ narrow search terms: “peripheral neuropathy” for any FDA-approved PI (bortezomib, carfilzomib, and ixazomib) and FXaI (apixaban, betrixaban, edoxaban, and rivaroxaban) as suspected, interacting, or concomitant in the current study. Reports with missing data for age, reporting year, gender, or age <18 years were excluded.

### Data Analysis

Different methods and analyses were performed to ensure the robustness and consistency of the results. In pharmacovigilance studies, the detection of the possible existence of DDIs is based on the assumption that a specific AE is reported more frequently when both drugs are used concomitantly compared to when they are used alone (Noguchi et al., 2019). Thus, reports were divided into three index groups: (Schlafer et al., 2017) reports of patients who received PI but did not receive FXaI; (Gavazzoni et al., 2018) reports of patients who received FXaI but did not receive PI; and (Cavaletti and Zanna, 2002) reports of patients who received both PI and FXaI. These index groups were compared with a reference group by comparing the rate of specific AEs for a given drug or drug class with the rate of the same effect (Noguchi et al., 2020). The patients in the reference group received neither PI nor FXaI.

A case/non-case disproportionality analysis was performed to compare the index and reference groups. The cases were reports of PN, whereas the non-cases were all other reports. A crude reporting odds ratio (ROR), along with a 95% confidence interval (CI), was calculated as a measure of the disproportionality between cases and non-cases for signal detection (van Puijenbroek et al., 2002) (Supplementary Table S2).

The signals were then refined with a dedicated correction to detect possible confounders present in the database. The crude RORs were adjusted for age, gender, reporting year and then calculated using unconditional logistic regression analysis (Noguchi et al., 2019). In constructing the logistic model, the PI, FXaI, and concomitant use of PI and FXaI (PIs*FXaIs) were coded, respectively. Furthermore, the terms for covariates, i.e., age, gender, and reporting year, were coded.

The logistic model was as follows:

Log(risk of the event) = β_0_+β_1_ PIs+β_2_ FXaIs+β_3_ PIs * FXaIs +β_4_A+β_5_G+β_6_Y, where A = age, G = gender, and Y = reporting year. In the analysis, PN was the dependent variable. Exposure categories were the use of FXaI or PI versus the use of none of these drugs. The use of FXaI and PI was added as an interaction term for the concomitant use of both drugs. Other covariates used in the analysis were the patient’s age, gender, and the year of AE reporting. β_3_ is the interaction coefficient. The exponentials of β_3_, exp(β_3_), are factors by which the ROR associated with combination therapy (FXaI * PI) exceeds that predicted by each drug of the pair alone. An additive effect of concomitant drug use occurs when the exp(β_3_) score is significantly different from 1. That is, the risk of AE for the use of combination drugs is greater than that predicted for each drug.

To test the consistency and reliability of drug interactions, we re-analyzed the data using multiplicative and additive models (Noguchi et al., 2019). The analysis provided a measure of the threshold for detecting a signal of DDIs. The multiplicative model assumed that the risk associated with a drug multiplies with the background risk, whilst the additive model assumed that the risk associated with a drug adds to the background risk. risk(drug_1_*drug_2_)/((risk(drug_1_)×risk(drug_2_))>1 and risk(drug_1_*drug_2_)-(risk(drug_1_)+risk(drug_2_))>0, respectively, indicate that the multiplicative and the additive models generate a drug interaction signal. That is, a positive interaction is present when the value (interaction term) > 0 (additive model) or 1 (multiplicative model) (Noguchi et al., 2020). Data management and analysis were performed using Microsoft Office Excel and IBM SPSS (version 13).

## Results

**FIGURE 1:**
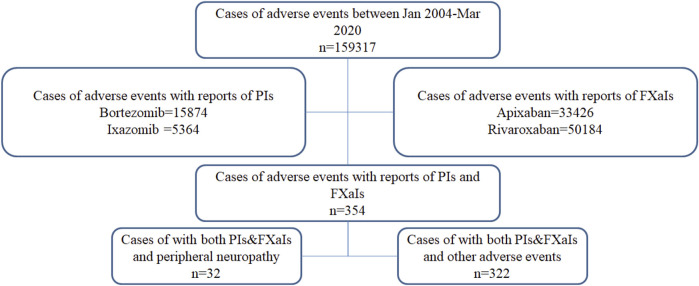
Data flow chart. FXaIs, Factor Xa Inhibitors; PIs, Proteasome Inhibitors.

After cleaning and extracting data from reports with complete age, reporting year, and gender information, a total of 159,317 reports (including 2,822 PN reports) were analyzed from the FAERS database (Figure 1). Due to the absence of PN reports of concurrent use of PI and FXaI, carfilzomib, betrixaban, and edoxaban were excluded for further analysis. The characteristics of the included patients are presented in Table 1; Supplementary Table S3. Most patients were ≥75 years (39.3%), with a median age of 71 ± 12.6 years. The treated patients were predominantly men, 52.7% (83,944/159,317), and from America, 67.3% (107,226/159,317). Most AE reports were severe, namely, resulting in “Initial or prolonged hospitalization” (48.4%), death (20.5%), or life-threatening events (5.2%). There was a significantly higher number of reports submitted during the study period. The characteristics of the patients in the index and reference groups are shown in Table 2.

**TABLE 1:** Characteristics of cases and non-cases.

	**Cases (%)**	**Non-cases (%)**
Total reports	2,822	156,495
Sex distribution		
Female	1,306 (46.3%)	74,067 (47.3%)
Male	1,516 (53.7%)	82,428 (52.7%)
Median age, years (SD)	67 (10.9)	71 (12.6)
Age distribution		
18–34	12 (0.4%)	1,831 (1.2%)
35–64	1,086 (38.5%)	44,128 (28.2%)
65–74	1,044 (37%)	48,665 (31.1%)
>75	680 (24.1%)	61,871 (39.5%)
Severity		
Initial or prolonged hospitalization	868 (30.8%)	76,295 (48.8%)
Disability	265 (9.4%)	3,346 (2.1%)
Life-threatening	101 (3.6%)	8,173 (5.2%)
Death	224 (7.9%)	32,435 (20.7%)
Geographical distribution		
America	1,645 (58.3%)	105,581 (67.5%)
Europe	770 (27.3%)	34,961 (22.3%)
Asia	213 (7.5%)	11,383 (7.3%)
Australia	48 (1.7%)	1,517 (1.0%)
Africa	3 (0.1%)	224 (0.1%)
Missing	143 (5.1%)	2,829 (1.8%)

SD, Standard Deviation.

**TABLE 2:** The characteristics of the patients in the index and reference groups.

	**Without PI**		**With PI**	
	**Without FXaI** ***n* = 55,599**	**With FXaI** ***n* = 82,643**	**Without FXaI** ***n* = 20,721**	**With FXaI** ***n* = 354**
Patient gender				
Female	24,975 (44.9%)	41,258 (49.9%)	8,991 (43.4%)	149 (42.1%)
Male	30,624 (55.1%)	41,385 (50.1%)	11,730 (56.6%)	205 (57.9%)
Median age, years (SD)	70 (10.9)	73 (13.7)	67 (11.3)	73 (9.6)
Patient age group (years)				
18–34	121 (0.2%)	1,542 (1.9%)	180 (0.9%)	0 (0%)
35–64	17,438 (31.4%)	19,428 (23.5%)	8,282 (40.0%)	66 (18.6%)
65–74	19,357 (34.8%)	22,870 (27.7%)	7,334 (35.4%)	148 (41.8%)
>75	18,683 (33.6%)	38,803 (47.0%)	4,925 (23.8%)	140 (39.5%)
Severity				
Initial or prolonged hospitalization	21,458 (38.6%)	46,332 (56.1%)	9,174 (44.3%)	199 (56.2%)
Disability	1,049 (1.9%)	2,065 (2.5%)	489 (2.4%)	8 (2.3%)
Life-threatening	2,002 (3.6%)	5,018 (6.1%)	1,227 (5.9%)	27 (7.6%)
Death	15,607 (28.1%)	13,064 (15.8%)	3,944 (19.0%)	44 (12.4%)

FXaI, factor xa inhibitors; PI, proteasome inhibitors; SD, Standard Deviation.

The crude RORs and 95% CIs for all comparisons are presented in Table 3 using a disproportionality method. PN was reported by 32 patients who used PI and FXaI concomitantly. When only PIs were used, 1,552 patients reported PN. When only FXaIs were used, 240 patients reported the occurrence of PN. The crude ROR for the use of PI alone was 4.430 (95% CI, 4.084–4.804), and the crude ROR for FXaI was 0.159 (95%CI, 0.138–0.184). An increase in PN reports for the concomitant use of PI and FXaI emerged (ROR = 5.437, 95%CI, 3.761–7.861), although not significantly higher than that found for PI alone.

**TABLE 3:** Reporting odds ratios and drug interaction approaches for proteasome inhibitors and factor Xa inhibitors.

**Drug-drug interaction of interest**	**Exposure**	**Cases**	**Non-cases**	**RORcrude (95%CI)**	**Additive model**	**Multiplicative model**
PI + FXaI	No PI, no FXaI	998	54,601	References		
	PI, no FXaI	1,552	19,169	4.430 (4.084–4.804)		
	FXaI, no PI	240	82,403	0.159 (0.138–0.184)		
	PI, FXaI	32	322	5.437 (3.761–7.861)	0.030	7.307

CI, confidence interval; FXaI, factor xa inhibitors; PI, proteasome inhibitors; ROR, reporting odds ratios.

Multivariate logistic regression was performed to identify the interaction between PI and FXaI by controlling for covariates (age, gender, and reporting year). Adjusted RORs for PN with PI treatment and FXaI treatment were 4.490 (95%CI, 4.136–4.874) and 0.194 (95%CI, 0.168–0.225), respectively (Table 4). The adjusted ROR of the interaction effect was 7.890 (95%CI, 5.321–11.698) between PI and FXaI, supporting the existence of an interaction (Table 4). The positive interaction signal was also supported based on additive/multiplicative models (Table 3).

**TABLE 4:** Adjusted reporting odds ratios of peripheral neuropathy events in cases with proteasome inhibitors and factor Xa inhibitors.

**Variable**		**Multivariate analysis**	
		**Adjusted ROR (95%CI)**	***p* value**
Patient gender			0.022
	Male	reference	
	Female	0.915 (0.848–0.987)	0.022
Patient age group (years)			<0.001
	18–34	reference	
	35–64	1.901 (1.065–3.393)	0.030
	65–74	1.936 (1.084–3.455)	0.025
	>75	1.471 (0.823–2.631)	0.193
Reporting year		0.953 (0.946–0.961)	<0.001
PI		4.490 (4.136–4.874)	<0.001
FXaI		0.194 (0.168–0.225)	<0.001
PI&FXaI		7.890 (5.321–11.698)	<0.001

CI, confidence interval; FXaI, factor xa inhibitors; PI, proteasome inhibitors; ROR, reporting odds ratios.

### Subset Data Analyses

**FIGURE 2:**
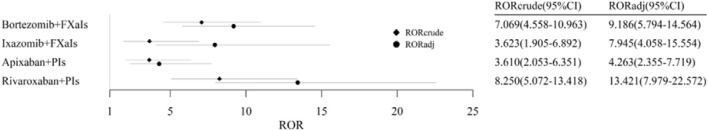
Reporting odds ratios of peripheral neuropathy in cases with factor Xa inhibitors and proteasome inhibitors. CI, Confidence Interval; FXaIs, Factor Xa Inhibitors; PIs, Proteasome Inhibitors; ROR, Reporting Odds Ratios.

Drug interactions between FXaI and PI were analyzed stratified by a single drug (bortezomib and ixazomib). Similar results were obtained. There was no interaction detected based on crude RORs. However, we investigated DDIs according to adjusted RORs and additive/multiplicative models. In addition, we failed to identify an interaction between apixaban and PI. The concomitant use of rivaroxaban and PI was associated with an increase in PN reporting based on crude ROR analysis. The results remained unchanged in multivariate logistic regression and additive/multiplicative models (Table 5; Figure 2). The results of DDIs between PI and FXaI evaluated by four approaches are summarized in Table 6.

**TABLE 5:** Disproportionality analyses and drug interaction approaches for the various drug combinations.

**Drug-drug interaction of interest**	**Exposure**	**Cases**	**Non-cases**	**RORcrude (95%Cl)**	**RORadj (95%Cl)**	**Additive model**	**Multiplicative model**
Bortezomib + FXaI	No Bortezomib, no FXaI	998	54,601	reference	reference		
	Bortezomib, no FXaI	1,351	14,322	5.161(4.746–5.612)	4.965(4.561–5.406)		
	FXaI, no Bortezomib	240	82,403	0.159(0.138–0.184)	0.186(0.161–0.215)		
	Bortezomib, FXaI	23	178	7.069(4.558–10.963)	9.186(5.794–14.564)	0.041	7.363
Ixazomib + FXaI	No Ixazomib, no FXaI	998	54,601	reference	reference		
	Ixazomib, no FXaI	220	4,983	2.415(2.081–2.803)	0.192(0.166–0.224)		
	FXaI, no Ixazomib	240	82,403	0.159(0.138–0.184)	2.949(2.506–3.469)		
	Ixazomib, FXaI	10	151	3.623(1.905–6.892)	7.945(4.058–15.554)	0.031	6.966
Apixaban + PI	No PI, no Apixaban	998	54,601	reference	reference		
	PI, no Apixaban	1,552	19,169	4.430(4.084–4.804)	3.999(3.686–4.338)		
	Apixaban, no PI	119	33,097	0.197(0.163–0.238)	0.259(0.213–0.315)		
	PI, Apixaban	13	197	3.610(2.053–6.351)	4.263(2.355–7.719)	-0.007	2.165
Rivaroxaban + PI	No PI, no Rivaroxaban	998	54,601	reference	reference		
	PI, no Rivaroxaban	1,552	19,169	4.430(4.084–4.804)	4.496(4.142–4.881)		
	Rivaroxaban, no PI	128	49,911	0.140(0.117–0.169)	0.167(0.139–0.202)		
	PI, Rivaroxaban	19	126	8.250(5.072–13.418)	13.421(7.979–22.572)	0.065	7.620

CI, confidence interval; FXaI, factor xa inhibitors; PI, proteasome inhibitors; ROR, reporting odds ratios.

**TABLE 6:** Summary of different method used to analyze drug-drug interactions.

**Drug–drug interaction of interest**	**RORcrude**	**RORadj**	**Additive model**	**Multiplicative model**
PI + FXaI	-	√	√	√
Bortezomib + FXaI	-	√	√	√
Ixazomib + FXaI	-	√	√	√
Apixaban + PI	-	-	-	√
Rivaroxaban + PI	√	√	√	√

FXaI, factor xa inhibitors; PI, proteasome inhibitors; ROR, reporting odds ratios.

– : no interaction detected.

√ : interaction detected.

## Discussion

To our knowledge, this is the first pharmacovigilance study to assess the drug interactions between PI and FXaI on PN. PI-induced PN is the most common chemotherapy complication well recognized in clinical practice. On the contrary, data for FXaI have rarely been reported. There are two unique findings from our analysis 1) the combined use of PI and FXaI was associated with a higher reporting of PN. This finding remained significant based on additive/multiplicative methods, and 2) the observed association was also consistent in analyses restricted to each specific PI (rivaroxaban, except apixaban) and FXaI (bortezomib and ixazomib).

Recently, several preclinical studies explored the mechanism of FXaI that aggravates chemotherapy-induced peripheral neuropathy (CIPN). High mobility group box 1 (HMGB1) facilitates pain signals and inflammation and plays a crucial role in the development of CIPN. HMGB1 activates the receptor for advanced glycosylation end-product (RAGE) and other receptors, causing hyperalgesia/allodynia in animal models. On the other hand, thrombomodulin (TM) promotes thrombin-induced degradation of HMGB1, thus preventing the development of CIPN and eliminating HMGB1-induced allodynia in rodents (Sekiguchi and Kawabata, 2021; Tsujita et al., 2021). Therefore, anticoagulants such as rivaroxaban or warfarin reduce the thrombin-dependent degradation of HMGB1 by TM, thus increasing plasma levels of HMGB1. The impact aggravates CIPN and cancels the anti-CIPN effect of TM in mice treated with oxaliplatin(10), contributing to neuropathy.

A recent placebo-controlled, double-blind, randomized study indicates that recombinant thrombomodulin, which directly binds to HMGB1 and improves its degradation by thrombin, has a preventive effect against oxaliplatin-induced PN (Kotaka et al., 2020). Two preclinical studies also found that recombinant thrombomodulin could prevent peripheral neuropathy induced by bortezomib and paclitaxel in rats and mice (Miyamoto et al., 2021; Tsubota et al., 2021). Tsubota et al. indicated that anticoagulant (argatroban) could promote bortezomib-induced peripheral neuropathy (BIPN) and cancel the anti-BIPN effect of recombinant thrombomodulin (Tsubota et al., 2021). The impact of anticoagulants on PN was dose dependent. A study found that three repeated oral administrations instead of a single administration of rivaroxaban reversed the anti-PN effect of thrombomodulin in animals treated with oxaliplatin (Tsubota et al., 2019). Thrombomodulin at 3 mg/kg could prevent and reverse BIPN in mice rather than at the 1 mg/kg dose (Tsubota et al., 2021).

In particular, LMWH, another anticoagulant that preferentially inhibits RAGE but not HMGB1 accumulation, can prevent and reverse HMGB1-dependent pain, including CIPN, in rodents treated with paclitaxel (Sekiguchi et al., 2018) or oxaliplatin (Tsubota et al., 2019). Therefore, this indicates that LMWH is theoretically a potential anticoagulant substitute for rivaroxaban.

Interestingly, our results show that PI interacts with rivaroxaban but not apixaban, which may be explained by a more stable and “mild” anticoagulation effect of apixaban. Apixaban appears to have more stable blood levels than rivaroxaban (Fralick et al., 2020). Increasing evidence reveals that rivaroxaban is associated with a higher risk of major bleeding despite its similar clinical effectiveness than apixaban (Noseworthy et al., 2016; Bonde et al., 2020). Further studies are needed to compare the safety profiles of rivaroxaban and apixaban when concomitant use of PI.

VTE is the second most common cause of death in cancer patients, in addition to the malignancy itself (Khorana, 2010). Individuals with multiple myeloma are at increased risk of developing VTE. The risk of VTE increases with immunomodulants (thalidomide, lenalidomide, and pomalidomide) and higher doses of corticosteroids. These drugs, along with PI, represent standard therapy for multiple myeloma (Lapietra et al., 2021). FXaI is recommended for the treatment of VTE in patients with multiple myeloma (Farge et al., 2019; Key et al., 2020). Although they are not routinely recommended to prevent VTE, FXaIs are becoming an effective candidate due to the advantages of not needing laboratory monitoring at routine doses and avoiding daily subcutaneous injections (Poulsen et al., 2012). Growing evidence shows that the use of FXaI effectively prevents VTE in patients with multiple myeloma on anticancer therapy compared to other anticoagulation regimens (Lapietra et al., 2021). As a result, more patients with multiple myeloma receive FXaI, indicating that the concomitant use of PI and FXaI is becoming common in clinical practice (Swan et al., 2018).

The findings of the pharmacovigilance analysis of potential DDIs between PI and FXaI may have significant clinical consequences, leading to poor adherence to treatment and clinical outcomes. Clinical practice on the concurrent use of PI and rivaroxaban should be based on the benefits and risks of PN. Our findings also provide evidence to improve the warnings included in prescribing these drugs.

DDIs are one of the leading causes of AE, considered a serious global health concern. Almost 500,000 serious medical complications occurred per year resulting from drug consumption, and some were fatal (Zhang et al., 2018). Therefore, identifying DDIs is a significant and pressing area of research. It is essential to provide knowledge for clinical applications, drug development, control of the medical cost associated with AE, and safeguarding patient well-being by acquiring information about possible AEs due to drug co-administration. Identifying DDIs using a publicly available FAERS data set is an efficient approach.

ROR is a technique that allows for adjustment through logistic regression analysis (van Puijenbroek et al., 1999; van Puijenbroek et al., 2000). Logistic regression can analyze interaction terms by controlling covariates (van Puijenbroek et al., 1999; van Puijenbroek et al., 2000). In our study, the crude ROR offered a rough indication of the interaction when PI was administered with FXaI, namely an upward trend, but not a significantly more frequent report of PN. Previous studies have indicated age and gender as key factors for PN (Cavaletti and Jakubowiak, 2010; Zgheib et al., 2018). Therefore, adjusted RORs with age, gender, and reporting year were calculated in the logistic regression analysis. The results suggested an increased risk of PN when PI and FXIa were used concomitantly.

The signal detected using FAERS should not be interpreted as an assumption of a causal relationship between drugs and clinical events, but rather as a suggestion of an association and serves as a starting point for further analysis. The limitations inherent in spontaneous reporting should be noted: 1) due to the voluntary nature of FAERS reporting, under-reporting, over-reporting, or missing information was inevitable (Michel et al., 2017); and 2) causal associations are not required before reporting AEs to the FAERS database. Limitations can confound the drug-event relationship. It is difficult to quantify the risk of interactions directly. Incomplete data and a lack of detailed patient information (e.g., co-medications and comorbidities) are often present in FARES. Therefore, it is difficult to perform a rigorous analysis of the potential impact of demographics. We excluded missing data records (low-quality reports), and the reports were cleaned prior to analysis despite these caveats. Since the incidence and background reporting rates in the spontaneous reporting system are relatively low, detecting DDIs is much more complicated than detecting drug-event combinations. Therefore, there was no distinction between suspected and unsuspected drugs to increase the sample size. However, several medications (such as carfilzomib) were excluded due to the lack of data availability. Lastly, we also do not know the doses of drugs used and laboratory values, such as blood coagulation and drug levels, to support a causal mechanism of interactions. As a result, to confirm our findings, additional robust epidemiological research is warranted to further test the hypothesis to draw conclusions that contribute to clinical practice.

Despite the limitations mentioned above, our analysis has a couple of strengths: 1) a narrow version of the selected SMQ term “peripheral neuropathy” was used to increase the specificity of the included AE reports, and 2) three validated methods controlled the main confounders (age, sex and reporting year), and sub-analyses were performed by specific PI and FXaI to strengthen consistency and robustness of possible drug interaction signals.

FAERS data suggest that there is more frequent reporting of PN when PI is administered concurrently with FXaI. Furthermore, the interactions remain in the analyses restricted to each specific PI (bortezomib and ixazomib) and FXaI (rivaroxaban, except apixaban). Physicians should exercise caution when prescribing these drug combinations. Potential remediation strategies can include switching FXaI to other anticoagulants or switching from rivaroxaban to apixaban.

## Data Availability

The raw data supporting the conclusions of this article will be made available by the authors, without undue reservation.
