# Supplementary material for: Peripheral Neuropathy During Concomitant Administration of Proteasome Inhibitors and Factor Xa Inhibitors: Identifying the Likelihood of Drug-Drug Interactions
Source: Front Pharmacol. 2022 Mar 14;13:757415. doi: 10.3389/fphar.2022.757415 (PMC8963930; doi:10.3389/fphar.2022.757415)
Supplement: Supplementary file 1 [file DataSheet1.docx]

**Supplement Table 1. Preferred terms associated with peripheral neuropathy in the narrow Standardized MedDRA Queries**

| Preferred terms |
| --- |
| Acute painful neuropathy of rapid glycaemic control |
| Acute polyneuropathy |
| Amyotrophy |
| Autoimmune neuropathy |
| Axonal neuropathy |
| Biopsy peripheral nerve abnormal |
| Central pain syndrome |
| Demyelinating polyneuropathy |
| Guillain-Barre syndrome |
| Ischaemic neuropathy |
| Loss of proprioception |
| Miller Fisher syndrome |
| Multifocal motor neuropathy |
| Myelopathy |
| Nerve conduction studies abnormal |
| Neuralgia |
| Neuritis |
| Neuronal neuropathy |
| Neuropathic muscular atrophy |
| Neuropathy peripheral |
| Notalgia paraesthetica |
| Peripheral motor neuropathy |
| Peripheral nervous system function test abnormal |
| Peripheral sensorimotor neuropathy |
| Peripheral sensory neuropathy |
| Polyneuropathy |
| Polyneuropathy chronic |
| Polyneuropathy idiopathic progressive |
| Radiation neuropathy |
| Sensorimotor disorder |
| Sensory disturbance |
| Sensory loss |
| Small fibre neuropathy |
| Tick paralysis |
| Toxic neuropathy |

**Supplement Table 2. Two by two frequency table for reporting odds ratios calculation**

|  | **"peripheral neuropathy" reported (cases)** | **"peripheral neuropathy" reported (non-cases)** |
| --- | --- | --- |
| Index group | a | b |
| Reference group | c | d |

Reporting Odds Ratio (ROR)=(a : c)/(b : d)

95% confidence interval＝e^ln (ROR)±1.96√(1^*^/a+^*^1^*^/b+^*^1^*^/c+^*^1^*^/d)^*

**Supplement Table 3. Demographic information of adverse events included from FAERS**

| **Characteristics** | **N (%)** |
| --- | --- |
| **Total reports** | 159,317 |
| **Patient gender** |  |
| Female | 75,373 (47.3%) |
| Male | 83,944 (52.7%) |
| **Median age (SD)** | 71(12.6) |
| **Patient age group (years)** | |
| 18–34 | 1,843 (1.2%) |
| 35–64 | 45,214 (28.4%) |
| 65–74 | 49,709 (31.2%) |
| >75 | 62,551 (39.3%) |
| **Severity** |  |
| Initial or prolonged hospitalization | 77,163 (48.4%) |
| Disability | 3,611 (2.3%) |
| Life-threatening | 8,274 (5.2%) |
| Death | 32,659 (20.5%) |
| **Reporting year** |  |
| 2004-2010 | 15,036 (9.4%) |
| 2011-2015 | 38,779 (24.3%) |
| 2016-2020 | 105,502 (66.2%) |
| **Geographical distribution** | |
| America | 107,226 (67.3%) |
| Europe | 35,731 (22.4%) |
| Asia | 11,596 (7.3%) |
| Australia | 1,565 (1.0%) |
| Africa | 227 (0.1%) |
| Missing | 2,972 (1.9%) |

SD, Standard Deviation
